# A multiplexed gas exchange system for increased throughput of photosynthetic capacity measurements

**DOI:** 10.1186/s13007-018-0347-y

**Published:** 2018-09-11

**Authors:** William T. Salter, Matthew E. Gilbert, Thomas N. Buckley

**Affiliations:** 1 School of Life and Environmental Sciences, Sydney Institute of Agriculture, The University of Sydney, Sydney, NSW Australia; 2 Department of Plant Sciences, University of California, Davis, Davis, CA 95616 USA

**Keywords:** Phenotyping, Photosynthesis, High-throughput, Gas-exchange, Photosynthetic capacity, *A*_max_

## Abstract

**Background:**

Existing methods for directly measuring photosynthetic capacity (*A*_max_) have low throughput, which creates a key bottleneck for pre-breeding and ecological research. Currently available commercial leaf gas exchange systems are not designed to maximize throughput, on either a cost or time basis.

**Results:**

We present a novel multiplexed semi-portable gas exchange system, OCTOflux, that can measure *A*_max_ with approximately 4–7 times the throughput of commercial devices, despite a lower capital cost. The main time efficiency arises from having eight leaves simultaneously acclimate to saturating CO_2_ and high light levels; the long acclimation periods for each leaf (13.8 min on average in this study) thus overlap to a large degree, rather than occurring sequentially. The cost efficiency arises partly from custom-building the system and thus avoiding commercial costs like distribution, marketing and profit, and partly from optimizing the system’s design for *A*_max_ throughput rather than flexibility for other types of measurements.

**Conclusion:**

Throughput for *A*_max_ measurements can be increased greatly, on both a cost and time basis, by multiplexing gas streams from several leaf chambers connected to a single gas analyzer. This can help overcome the bottleneck in breeding and ecological research posed by limited phenotyping throughput for *A*_max_.

**Electronic supplementary material:**

The online version of this article (10.1186/s13007-018-0347-y) contains supplementary material, which is available to authorized users.

## Background

Leaf gas exchange traits are important in plant breeding, physiology and ecology research. The ability to measure such traits using mass produced, field portable gas exchange systems has made these systems a staple of many laboratories, and their impact on scientific progress cannot be overstated. However, these systems were designed to maximize portability and flexibility, and as a result, they are not optimized for maximal throughput in phenotyping studies. For example, because leaves can take around 12–15 min [1] to acclimate to saturating CO_2_ and light before measuring photosynthetic capacity (light-and CO_2_-saturated maximum net CO_2_ assimilation rate, *A*_max_), throughput cannot exceed 4–5 measurements per hour with a single-chamber commercial system. Increasing throughput thus requires the purchase of a large number of units. These constraints on throughput are compensated by the flexibility and portability of commercial systems, which can rapidly change chamber conditions at the user’s command and can be carried by hand to measure plants in situ, even in difficult terrain. However, because that flexibility is expensive to engineer and implement, it is sub-optimal with respect to throughput and cost in phenotyping studies that do not require such flexibility.

Alternative high-throughput approaches for studying gas exchange, though highly promising, are typically indirect (e.g., NDVI, hyperspectral imaging, chlorophyll fluorescence, IR thermography), and nevertheless require calibration and validation against direct gas exchange measurements. Direct systems are often only practical for application to plants grown in small growth containers suitable for mechanized measurement (e.g., conveyor based systems, gravimetric systems) (for review of current high-throughput phenotyping technology see [2].

**Fig. 1:**
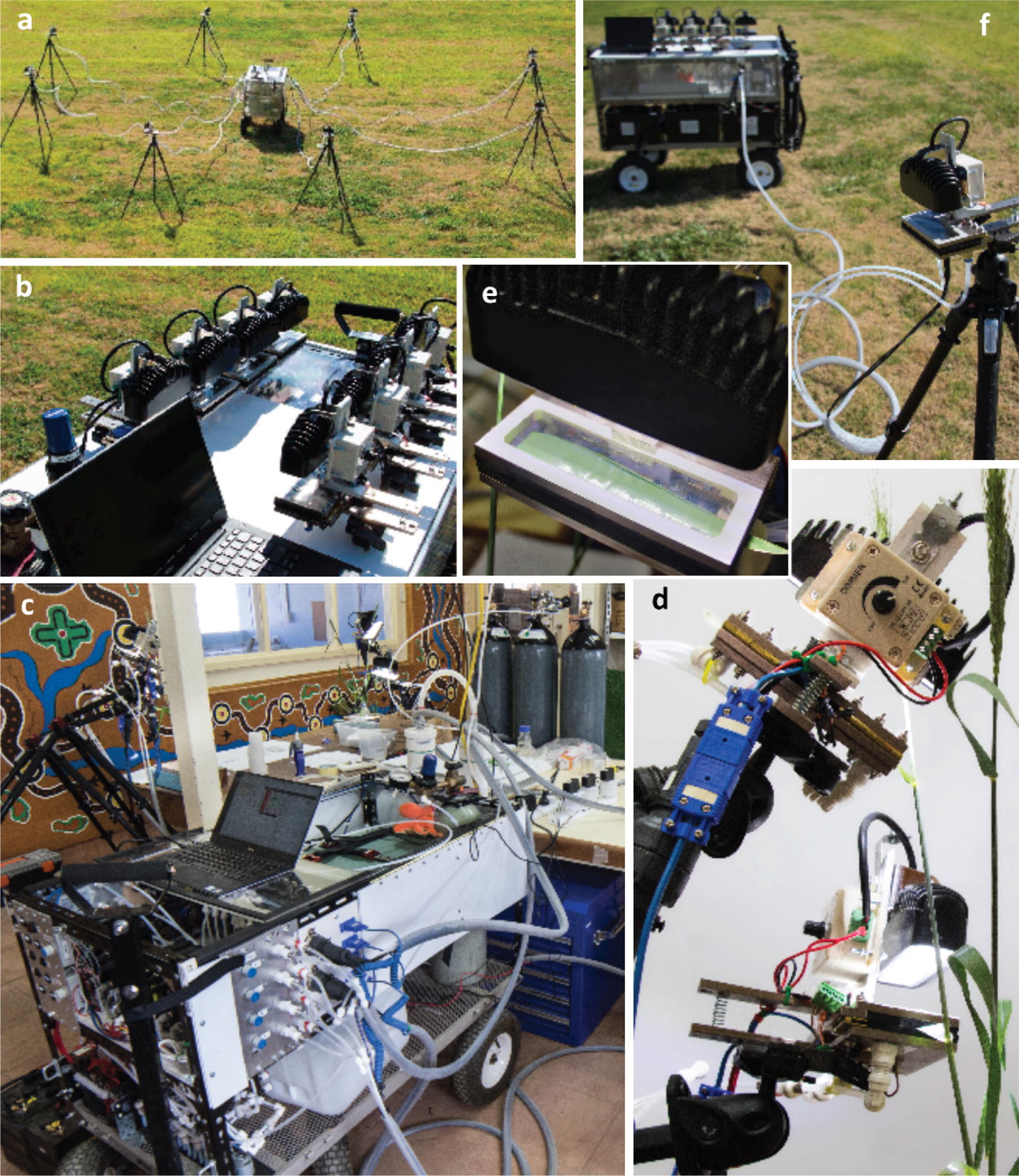
Photographs of OCTOflux. Clockwise from top left: **a** Mothership (center) connected to eight chambers on tripods. **b** Top deck of mothership, with chambers docked. CO_2_ regulator is visible at lower left. **c** OCTOflux in operation in the laboratory in Narrabri. Four chambers are visible at top left and top center, on tripods. Gas, power, data and thermocouple connections between chambers and the mothership are at lower center. **d** Two OCTOflux chambers measuring the flag leaf and second leaf of a single wheat tiller in the laboratory. **e** Wheat leaf in an OCTOflux chamber, below its LED light source (black object at top center). **f** An OCTOflux chamber connected to the mothership in the field (three truck batteries are visible on the lower level of the mothership)

In this study, we describe a semi-portable gas exchange system, OCTOflux (Fig. 1), designed to maximize throughput of *A*_max_ measurements in field crops. Leaves are enclosed in eight chambers sequentially and exposed to saturating light and CO_2_ > 4000 ppm, as needed to ensure that variations in stomatal conductance do not influence measurements. Traditional CO_2_ response curves and modeling can be used in separate validation experiments to ensure that *A*_max_ is not substantially reduced by triose phosphate utilization at these high CO_2_ concentrations. Each chamber’s sample gas stream is channelled through an infrared gas analyzer for 60 s after acclimation is complete. CO_2_ is injected into a pressurized air stream from a tank using a mass flow controller, and reference gas composition is stabilized using a large buffer volume (~ 20 L). OCTOflux achieved an average throughput of 16.7 values of *A*_max_ per hour in a trial campaign using wheat; the total capital cost was ~ USD $31,000. Below, we describe the system in detail, present sample output data, and discuss modifications to further enhance throughput.

## Results

**Fig. 2:**
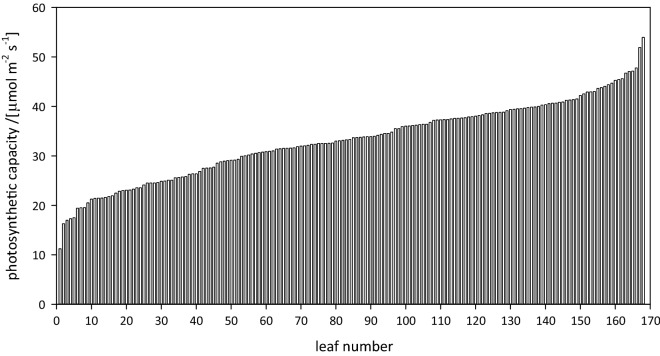
Processed output from 1 day of OCTOflux measurements: 168 measurements of photosynthetic capacity (*A*_max_), each on a different leaf of wheat. These measurements were completed in approximately 7.5 h

We completed 165 measurement cycles (1320 *A*_max_ measurements) over 12 days. Measurement cycle length averaged 28.7 ± 5.8 min (mean ± SD) and ranged from 17 to 50 min, with 90% of cycles taking between 21 and 40 min. Much of this variation arose from differences in photosynthetic acclimation time, and the rest resulted from logistical factors unrelated to OCTOflux. Sample data from a typical day (168 individual measurements of *A*_max_) is shown in Fig. 2.

### Functional characteristics

**Fig. 3:**
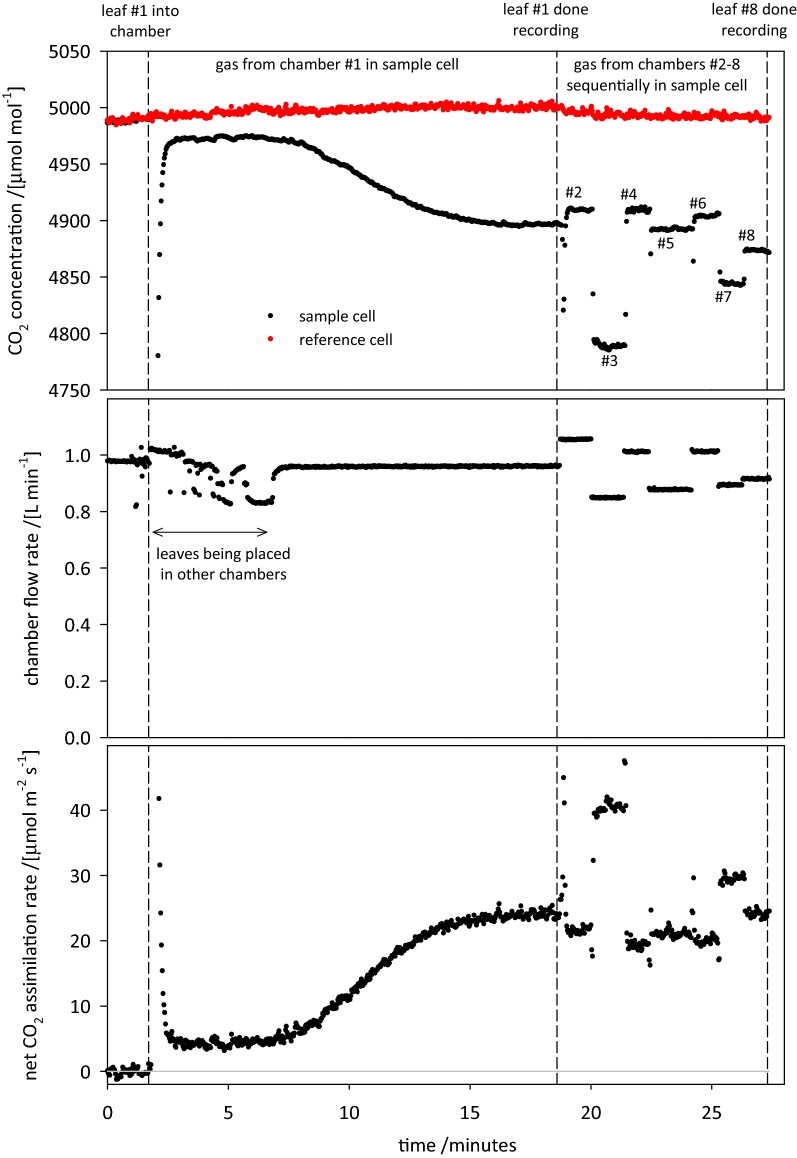
Sample OCTOflux measurement cycle. A leaf is placed in chamber #1 at the time indicated by the first dashed line; leaves are placed in the other seven chambers for the next several minutes (as evidenced by fluctuations in chamber flow rate (b). After around 16 min, the assimilation rate for leaf #1 has stabilized (second dashed line), and the solenoid valves are adjusted to direct sample gas from chamber #2 through the IRGA sample cell. This is repeated over the next 7 min for the remaining chambers. The cycle is complete when the 8th leaf is done recording

A typical measurement cycle is shown in Fig. 3. The trace for *A* begins with a mixing lag caused by small transient fluctuations in total system flow (and hence in the ratio of CO_2_ injection flow rate to total flow) while leaves are being placed in chambers. After 3 min, this mixing lag has passed. From the start of the recording until 18.6 min, gas from chamber #1 was flowing through the IRGA (infra-red gas analyser) sample cell, showing a typical sigmoidal acclimation response of *A* to saturating light. After that response stabilized, sample gas from each of the other seven chambers was sent through the analyzer sequentially, for 1 min each.

Chamber flow rates were set at approximately 1 L min^−1^ but varied among chambers due to minor differences in tubing length between the mothership and chambers. Flow rates also fluctuated while leaves #2–8 were being placed in chambers, due to the reduction in upstream gas pressure caused by temporarily opening each chamber to put a leaf into it (e.g., Fig. 3).

### Empty chamber test

**Fig. 4:**
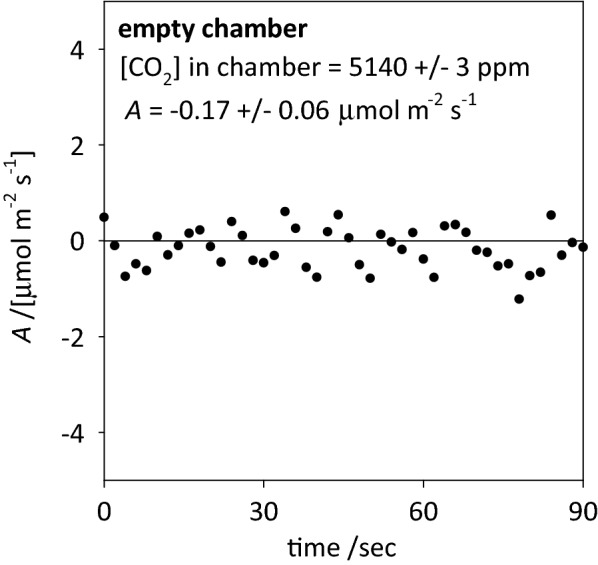
Net CO_2_ assimilation rate calculated for a chamber not containing a leaf, with [CO_2_] in the chamber of 5140 ppm and [CO_2_] outside the chamber at approximately 400 ppm

The value of *A* calculated with no leaf in the chamber, at a chamber [CO_2_] of 5140 ppm, averaged − 0.17 ± 0.06 μmol m^−2^ s^−1^ (mean ± SE) over 90 s (Fig. 4). Because we used a 40-s average of *A*_max_ in normal operating conditions, we computed the mean and standard error of *A*_max_ for every contiguous 40-s interval within the 90-s empty chamber test; the resulting mean and SE within these 40-s intervals varied between − 0.27 ± 0.10 to − 0.06 ± 0.09 μmol m^−2^ s^−1^ and averaged − 0.14 ± 0.10 μmol m^−2^ s^−1^. These results show that diffusion across the chamber gaskets was an insignificant component of measured *A*.

Leaks can also occur due to imperfect sealing around leaf midribs. We detected such leaks by noting when chamber flow rate was greater with leaves in the chamber than without, and in such cases we sealed the leak using clear silicone gap-filling compound. Leak sealing generally had no effect on calculated gas exchange rates, however, indicating that the leaks were predominantly advective and that the chamber air was thoroughly mixed (which together would ensure that leaked air had the same composition as air exiting the sample outlet, and thus did not affect gas exchange calculations).

### Temperature responses

**Fig. 5:**
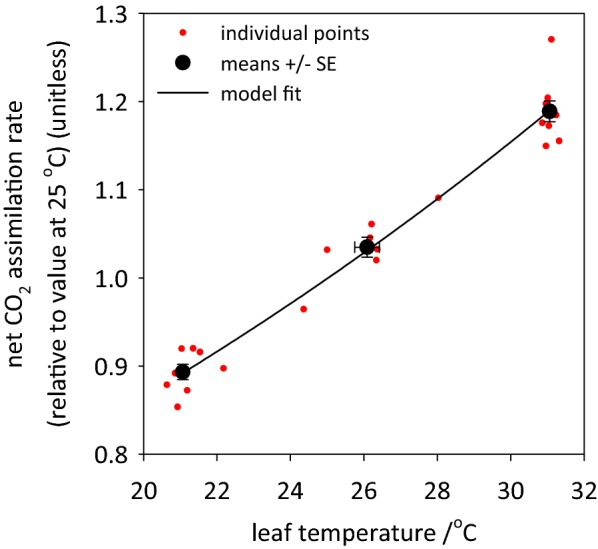
Temperature responses of *A*_rel_ (*A*_max_ expressed relative to each leaf’s value at 25 °C). Red symbols = individual points (3 per leaf); solid black symbols = means ± SE within each temperature bracket; solid line = regression of *A*_rel_(*T*) versus *A*_rel_(25)·exp(*bT*)

*A*_max_ relative to its value at 25 °C [*A*_rel_(*T*) = *A*_max_(*T*)/*A*_max_(25)] was exponentially related to leaf temperature: *A*_rel_(*T*) = 0.485977·exp(0.028831·[*T*_leaf_/°C]) (residual *df* = 25, *r*^2^ = 0.963; Fig. 5).

### Validation of TPU-limited *A*_max_ at high CO_2_ in relation to *A* versus *c*_*i*_ parameters

**Fig. 6:**
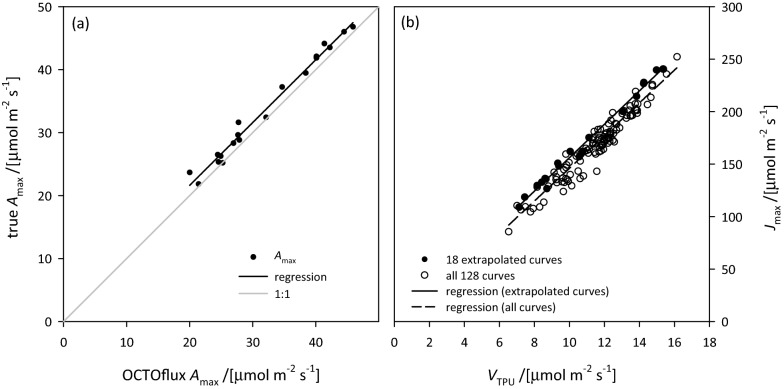
Relationships between **a** true *A*_max_ (value under electron transport limited conditions) and OCTOflux *A*_max_ (value at high CO_2_ under TPU-limited conditions, estimated by extrapolating to high *c*_i_ the Busch et al. [12] model for TPU-limited *A* fitted to *A* versus *c*_i_ curve data for 18 *A* versus *c*_i_ curves for which data were adequate for this purpose), and **b** *V*_TPU_ and *J*_max_ estimated from *A* versus *c*_i_ curves for the 18 curves indicated in **a** (solid symbols) and for all 128 *A* versus *c*_i_ curves measured (open symbols). In both **a** and **b**, regressions for the 18 curves are shown with solid lines (a: *y* = 0.9968*x* + 1.7064, *r*^2^ = 0.9841; b: *y* = 0.0622*x* + 0.298, *r*^2^ = 0.9911); in **b**, a regression for the open symbols is shown with a dashed line (*y* = 0.0597*x* + 1.3857, *r*^2^ = 0.9301)

Because triose phosphate utilization (TPU) can limit *A*_max_ at high CO_2_, we investigated whether such an effect would have an influence on results measured at saturating CO_2_ in wheat. We obtained 19 *A* versus *c*_i_ curves that had depressions in *A* at high CO_2_, and thus could be used for modelling the decline in *A* with increasing *c*_i_ under TPU-limited conditions. Among these curves, the value of *A*_max_ projected at *c*_i_ = 5000 ppm was proportional to the true *A*_max_ under electron transport limited conditions (*A*_max_[OCTOflux] = 0.9968·*A*_max_[e-tpt] + 1.7064; *r*^2^ = 0.9841, *n* = 18; Fig. 6a), and *V*_TPU_ was proportional to *J*_max_ (*V*_TPU_ = 0.0622·*J*_max_ + 0.298; *r*^2^ = 0.9911, *n* = 18; Fig. 6b, solid symbols). Among all *A* versus *c*_i_ curves (including those for which TPU-limited points were inadequate to model the decline in *A* with increasing *c*_i_), *V*_TPU_ was also proportional to *J*_max_, and with a slope similar to that found among the 18 curves described above (*V*_TPU_ = 0.0597·*J*_max_ + 1.3857, *r*^2^ = 0.9301, *n* = 128; Fig. 6b, open symbols).

## Discussion

The OCTOflux system was able to measure *A*_max_ with far greater throughput than would have been possible using a single commercial system, and at far lower cost than possible using several commercial systems to match OCTOflux’s throughput. We achieved an average throughput of 16.7 measurements of *A*_max_ per hour—4.4 times greater than the 3.8 measurements per hour possible with a single-chamber commercial system, given the mean time for acclimation of *A*_max_ to saturating PPFD (13.8 ± 0.4 min to reach 95% of *A*_max_; mean ± SE, n = 131 leaves; data not shown) and allowing 2 min per measurement to enclose a leaf in the chamber, then remove it and measure its area (in cases where the leaf does not completely fill the chamber of a commercial system). The acclimation delay could in theory be avoided by having many leaves acclimate under saturating PPFD in a system outside of the IRGA chamber for 15–20 min, although this would generate some expense and workload, and it would be necessary to ensure that the external PPFD was at least as great as the chamber PPFD to avoid any subsequent acclimation delay. Alternatively, one could operate four or five single-chamber systems concurrently, but this would increase the capital cost dramatically. For example, a Li-Cor Li-6800 costs ~ USD $50,000 at the time of writing, so achieving OCTOflux’s throughput would require at least $200,000 in capital expenditure. By comparison, OCTOflux cost approximately USD $31,000 to construct, giving roughly seven times greater throughput per unit capital cost.

**Fig. 7:**
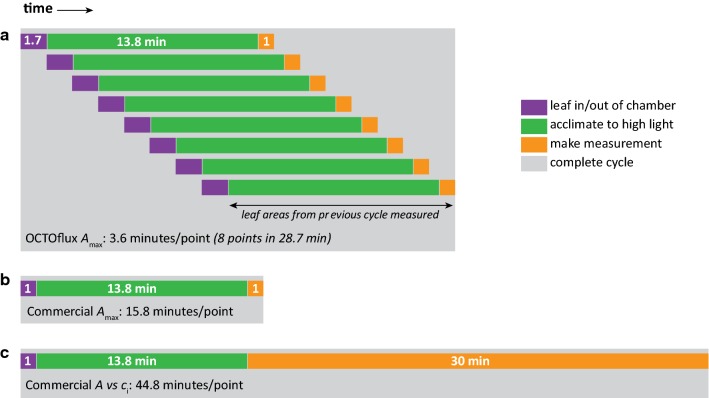
Diagram of workflow for three methods of measuring photosynthetic capacity (*A*_max_) by gas exchange (**a** *A*_max_ measured with OCTOflux; **b** *A*_max_ measured with a commercial single-chamber system; **c** *A*_max_ and other parameters inferred from *A* vs *c*_i_ curves measured with a commercial single-chamber system), illustrating the reason for OCTOflux’s greater throughput per unit time: namely, in *A*_max_ measurements with single-chamber commercial systems **b**, the gas analyzer sits idle while the leaf acclimates to saturating light (which took an average of 13.8 min in the present study). Traditional *A* versus *c*_i_ curves **c** take much longer still (approximately 30 min to complete measurements, vs ~ 1 min to complete a spot measurement of *A*_max_), although they do provide much more information than *A*_max_. Note in **a** that measurement of leaf areas from one OCTOflux measurement cycle can be completed during the idle time of the next cycle

There are two main reasons for OCTOflux’s greater throughput. First, allowing multiple leaves to simultaneously acclimate to saturating light and CO_2_ reduces the IRGA’s downtime (Fig. 7). This efficiency could be further enhanced by adding more chambers. Throughput (*t*, measurements per unit time) is given by

1 $$t = \frac{n}{{n\left( {i + p} \right) + a + r}},$$

where *n* = number of chambers, *i* = time required to put each leaf into a chamber, *p* = time to remove each leaf from the chamber, *a* = time for each leaf to acclimate to chamber conditions, and *r* = time allowed to record stable gas exchange for each leaf. Because Eq. 1 is a monotonically increasing function of *n*, adding chambers always increases throughput. For example, given the values for *t*, *a*, *r* in this study (16.7 leaves per hour, 13.8 and 1.0 min, respectively, giving *i* + *p* = 1.7 min), the throughput with 16 chambers would be 22.5 measurements per hour, or six times the throughput of a single-chamber system. Realistically, space constraints would eventually limit the number of chambers that can practically be operated. For measurements where acclimation time is short (e.g., 2 min) the eight chamber OCTOflux would still have a throughput advantage over commercial single chamber systems (on the order of twice the throughput). In short, although commercial gas exchange systems unquestionably have many advantages over OCTOflux (see *Limitations of OCTOflux, and potential extensions and improvements*, below), the OCTOflux approach—multiple chambers and a single IRGA—can greatly increase throughput per unit capital cost and per unit time in studies involving gas exchange measurements that require substantial in-chamber acclimation time.

### Comparison to high-throughput methods for phenotyping photosynthetic traits

High throughput phenotyping platforms (HTPPs) have been heralded as the future of plant breeding and are already changing the nature of breeding research [3]. Most HTPPs are based on indirect canopy measurements such as thermal imagery, hyperspectral reflectance, NDVI, LIDAR and infrared thermography (for estimating transpiration), which offer orders of magnitude greater throughput than traditional methods. For example, a rotocopter drone fitted with imaging sensors could phenotype an entire field within 1 h [4]. HTPPs have also been established in glasshouses or controlled environment facilities, where sensors can be larger and more powerful and can continually monitor physiology, and where potted plants can be moved around using automated conveyor systems and weighed to monitor growth and water use.

Current HTPPs have two major limitations. First, some HTPP systems, notably automated systems, are prohibitively expensive, which limits their potential for widespread phenotyping in different environments. Second, and more generally, HTPPs measure indirect proxies for *A*_max_, with calibrations that can vary across plant species, genotypes, developmental stages and field conditions [5]. Thus, such proxies require intensive validation against direct gas exchange measurements. Field validation has thus far been limited to small sets of genotypes/species with limited replication, due to throughput constraints of single-chamber gas exchange systems [6–8]. Traits measured using indoor HTPPs on potted plants may differ greatly from those measured in field conditions [9], which questions their applicability in agronomic or ecological contexts. OCTOflux can facilitate the validation of field-based HTPPs.

### Comparison to measurements based on photosynthetic *A* versus *c*_*i*_ curves

The standard method for measuring photosynthetic capacity in plant physiology has for many years been to measure the response of *A* to *c*_i_, fit a biochemical model [10] and extract the resulting parameters of photosynthetic capacity (*V*_cmax_ and *J*_max_). One value of that approach is that *A* vs *c*_i_ curves are independent of stomatal effects (provided stomatal conductance is not spatially heterogeneous or “patchy”). By measuring *A* at very high ambient CO_2_ (> 4000 ppm), at which stomata no longer influence *A*, OCTOflux has the same benefit. However, OCTOflux provides less information than an *A* vs *c*_i_ curve: in fact, the value of *A*_max_ measured by OCTOflux represents a value limited by the rate of triose phosphate utilization (*V*_TPU_) or RuBP-regeneration (*J*_max_). Our *A* versus *c*_i_ curve data showed that *V*_TPU_ is an excellent predictor of *J*_max_, and that the CO_2_-saturated value of *A*_max_ reported by OCTOflux is an excellent predictor of the “true” *A*_max_, which occurs at the point of transition between electron transport limitation and TPU limitation: OCTOflux *A*_max_ was linearly related to true *A*_max_ with a slope of 0.9968 and an *r*^2^ of 0.9841 across 18 leaves ranging in *A*_max_ from 20 to 46 μmol m^−2^ s^−1^). Thus, OCTOflux provides a faithful estimate of photosynthetic capacity as estimated from *A* versus *c*_i_ curves. Despite providing less information than *A* versus *c*_i_ curves, OCTOflux-based *A*_max_ estimates have the advantage that they do not depend on estimation of *c*_i_ (which is more uncertain than *A*_max_ because it depends on the ratio of *A* to stomatal conductance, and thus compounds errors in CO_2_ and H_2_O exchange and leaf temperature measurement), nor on estimation of mesophyll conductance, *g*_m_, which determines the relation of chloroplastic CO_2_ concentration (*c*_c_) to *c*_i_. We suggest that, in phenotyping studies in which the detailed information provided by *A* versus *c*_i_ curves in conjunction with *g*_m_ estimation is not needed, OCTOflux provides a sound and efficient alternative.

### Limitations of OCTOflux, and potential extensions and improvements

Just as single-chamber systems are not optimized for throughput, OCTOflux is not optimized for many experimental situations. First, the system is too large for use in rough or remote terrain. Second, it has no humidity control, although this could be rectified by adding a high-capacity humidifier and a system for mixing dry and humid air. Third, it lacks leaf temperature control. Peltier temperature controllers could be added to each chamber, though this would greatly increase power demand, reducing the feasibility of operating the system under field conditions without AC power. Fourth, many commercial systems can measure chlorophyll fluorescence parameters, but adding such capacity to multiple chambers in an OCTOflux-type system would greatly increase cost and complexity. Finally, using a large buffering volume to stabilize reference gas composition prevents rapid changes in [CO_2_] needed for CO_2_ response curves. This could be partially rectified by eliminating the buffer volume and extending the recording time for measurements of *A*_max_ to average over the fluctuations in CO_2_ that would result.

As currently configured, use of OCTOflux in the field is limited mainly by power and gas supplies. The large air cylinders used in the lab would limit the system’s mobility in the field; they could be replaced by a pump and scrubbers to remove H_2_O and CO_2_ from ambient air. The current design has room for four 12-V truck batteries on the lower shelf, which was adequate for 6 h of field operation in an earlier, pump-driven prototype with a different IRGA (which consumed less power than the Li-7000). However, temperature control would be more important under field conditions, greatly increasing power requirements. Possible solutions include towing a second garden cart filled with truck batteries, or using a portable electric generator to power the system. Whether such solutions are feasible would depend on the particular field situation; for example, they would pose little challenge for phenotyping row crops on relatively level ground.

We plan to modify OCTOflux in several ways to improve its performance, control and throughput. Some of these improvements were described earlier, including adding Peltier temperature controllers to each chamber and adding humidity control. Adding more chambers and using shorter chamber-IRGA connections would increase throughput and reduce settling time. Using a pre-mixed air tank with the desired reference gas CO_2_ composition would reduce noise in the IRGA CO_2_ differential, improving resolution and reducing measurement averaging time.

## Conclusion

Multiplexing gas streams from eight leaf chambers connected to a single IRGA increases the throughput for *A*_max_ measurements approximately four- to seven-fold on a time or capital cost basis, respectively, and further increases in throughput are possible using even more chambers. This approach can help overcome the bottleneck in breeding and ecological research posed by limited phenotyping throughput for physiological traits.

## Methods

### OCTOflux overview

The OCTOflux system consists of eight leaf chambers connected to a “mothership,” built on a 1.2 × 0.6 m garden cart modified with steel framing to produce three levels (Fig. 1). The mothership houses a differential CO_2_/H_2_O infrared gas analyzer (Li-7000, Li-Cor, Lincoln, NE), and numerous other components described below. The system is designed to be used either in the field or in the laboratory, although its utility in the field is limited by the lack of chamber temperature control in the current implementation. In this study, we operated the system in an air-conditioned laboratory to reduce variation in leaf temperature, increase operating time by using AC power for some components, and increase throughput by eliminating the need to move the system (including eight tripods and chambers) between plots in the field and to enable real-time processing of leaf images and measurement cycle metadata on a laboratory computer.

### Leaf chambers

Each chamber is made from custom machined, nickel-plated aluminum parts, and includes four small mixing fans (UB3F3-500, SUNON, Kaohsiung City, Taiwan), a type-T fine-wire (36 gauge) thermocouple (TT-T-36-100, OMEGA Engineering, INC., Norwalk, CT, USA) and an LED light source (WL-18 W-O60, Super Bright LEDs, Inc., St. Louis, MO, USA) situated above a propafilm window. These chambers were designed for wheat leaves, enclosing an area of up to 11 × 5 cm, with an internal volume of approximately 90 cm^3^.

### Flow pattern and operational principle

**Fig. 8:**
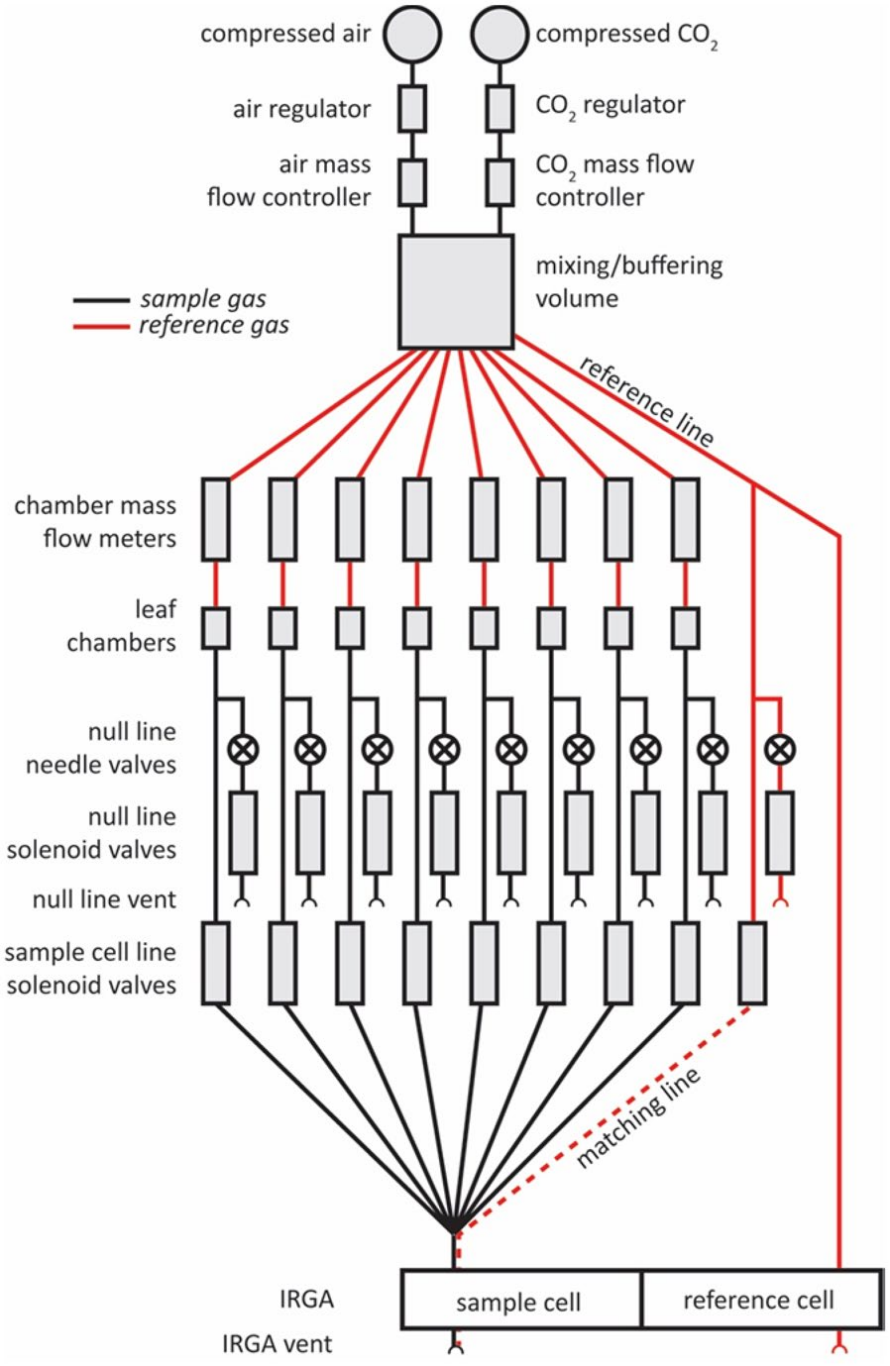
Functional schematic of OCTOflux system. Open semicircles represent atmospheric vent points. CO_2_ is injected into a stream of pressurized air, mixed in a buffering volume and distributed to eight chambers and a reference line. The return line of each chamber is either vented through its null line vent or directed through the IRGA sample cell using solenoid valves

Compressed air is injected into a buffer volume through a dual-stage regulator and a mass flow controller (MFC; FMA5420, Omega Engineering, Inc.) (Fig. 8). CO_2_ is injected into the buffer through a regulator and MFC (FMA5412, Omega). Buffer air is mixed with a 12 V CPU fan (PF40281B1-000U-G99, Sunon, Brea, CA, USA). Air exits the buffer through nine separate 1/4″ o.d. tubes (one per leaf chamber + a reference line). Each chamber line goes through a mass flow meter (MFM; 822-13-0D1-PV1-V1 MFM, Sierra Instruments, Monterey, CA, USA) and then through 5 m of 1/4″ tubing to a leaf chamber and back (3/16″ i.d. for tubing going to the chambers, and 1/8″ i.d. for tubing returning from chambers), before splitting to two one-way direct acting solenoid valves (2ACK-1/4, WIC valve, San Jose, CA, USA), termed the “sample” and “null” valves. The null valves vent to the atmosphere and the sample valves lead to the IRGA sample cell. The reference stream splits three ways: one line goes to the IRGA reference cell and the other two form sample and null lines, like the chamber lines. Flow rates of 0.5–2.0 L min^−1^ were possible with this system, representing turnover times of approximately 7.5–30 s; a flow rate of 1 L min^−1^ (turnover time 15 s) was typical in operation.

At any given time, only one of the nine sample solenoid valves is open, so only one gas source (one of the eight chamber lines or the reference line) flows into the IRGA sample cell; that line’s null valve is closed, and the null valves for the other eight lines are open. To “match” the IRGA, the reference line’s sample valve is opened and all chamber sample valves are closed, so that the same (reference) gas flows through both IRGA cells. Each null valve is preceded in the flow path by a needle valve, which enables the user to match flow resistances among lines to prevent changes in chamber flow rate when switching between chamber lines. Whenever the gas source entering an IRGA cell is changed, it takes approximately 15 s to turn over the air in the IRGA cell (for a chamber line flow rate of 1 L min^−1^), after which the IRGA CO_2_ and H_2_O differentials can be used to calculate current gas exchange rates [11].

### Data acquisition and processing and system control

The system is interrogated and controlled by a Microsoft Excel file that uses Visual Basic for Applications (VBA) to interface with the IRGA, a data acquisition board and a relay control board (USB-2416-4AO and USB-ERB24, Measurement Computing Corporation, Norton, MA, USA) in real time via Visual Basic functions in a DLL (see Additional files 1 and 2 for the Excel file and VB code, respectively). The Excel file uses Forms Controls to manipulate the system and real-time graphs of the data. The relay control board drives solid-state relays (DC60S3-B, Crydom, San Diego, CA, USA) that control voltage supplies to the solenoid valves. Control and measurements are performed every 2 s. We emphasize that the general approach presented in this study could be implemented using any suitable data acquisition and control system.

### Operational procedure

A typical OCTOflux cycle of eight *A*_max_ measurements involves five stages: enclosing leaves in chambers (~ 2–5 min total for eight leaves), waiting for *A* to stabilize at *A*_max_ (~ 8–25 min), sequentially routing each chamber’s sample gas through the analyzer to record its stable *A* value (7 min; 40–60 s per chamber), removing leaves from chambers (~ 1–2 min), and photographing leaves and measuring the leaf area enclosed in the chamber (~ 5 min). We performed the last (photographing) stage of each measurement cycle during the acclimation stage of the following cycle.

### Validation of *A*_max_ measurement

At very high CO_2_ concentration such as measured by OCTOflux, net CO_2_ assimilation rate can be limited by triose phosphate utilization (TPU; i.e., photoassimilate export) rather than by the capacities for RuBP carboxylation or electron transport (*V*_cmax_ and *J*_max_, respectively), and it is unknown whether the maximum TPU rate (*V*_TPU_) is strongly correlated with *J*_max_. Furthermore, *A* can decline with increasing intercellular CO_2_ concentration (*c*_i_) when TPU is limiting, so TPU-limited *A*_max_ can be lower than the “true” (electron transport-limited) *A*_max_. Busch et al. [12] recently showed that the decline in *A* with [CO_2_] under TPU-limited conditions is caused by quenching of non-photosynthetic CO_2_ assimilation (via C incorporation into amino acids in the photorespiratory cycle). However, this quenching saturates as glycine and serine export rates approach biochemical limits, so that *A* does not continue to decline indefinitely as *c*_i_ increases.

To validate the interpretation of OCTOflux’s high-*c*_i_ *A*_max_ measurement in relation to electron transport-limited *A*_max_, we measured *A* vs *c*_i_ curves in 128 leaves of 30 wheat genotypes. The curves were made with two recently calibrated IRGAs (GFS-3000; Heinz Walz GmbH, Effeltrich, Germany), by changing *c*_a_ in 13 steps (400, 50, 100, 150, 250, 350, 400, 600, 800, 1000, 1200, 1500 and 2000 µmol mol^−1^) over 45 min using a PAR of 2000 µmol m^−2^ s^−1^ and a temperature of 25 °C. These response curves used a 4 × 2 cm leaf chamber, a leaf to air vapor pressure difference of 1.5 ± 0.2 kPa (mean ± SD) and a chamber flow rate of 750 μmol s^−1^. We then fitted the Farquhar et al. [10] photosynthesis model to each curve, using the ‘plantecophys’ package in R (bilinear fitting method with TPU limitation estimate; [13], to estimate *V*_cmax_, *J*_max_ and *V*_TPU_. We then fitted the model proposed by Busch et al. [12] to the TPU-limited portion of *A* versus *c*_i_ curves in cases where enough data were available (4 or 5 TPU-limited points) and *A* was unambiguously declining with increasing *c*_i_ (which we defined as no more than one deviation from a monotonically declining relationship among the 4–5 points), and used the model to extrapolate *A* to its value at 5000 ppm to estimate the value of *A*_max_ that OCTOflux would give for that leaf.

### Calibration

We calibrated the IRGA for H_2_O using dry air (scrubbed using Drierite) and using ambient air, both also measured with a chilled mirror dewpoint hygrometer (Dew-10, General Eastern, GE, Billerica, MA, USA), and for CO_2_ using CO_2_-free air (scrubbed using soda lime) and reference tanks of 360 and 1190 ppm. Previous calibrations showed negligible span drift over time. We matched the IRGA sample and reference cells using reference air several times daily. We found that match drift was negligible with this analyzer if ambient temperature was stable and gas concentrations did not differ greatly between successive measurements, provided the instrument was warmed up for ≥ 2 h. During this study, we kept the analyzer running 24 h per day.

We calibrated the MFMs and MFCs by first calibrating one MFC volumetrically (recording the time required for air flow at each of several different flow rates to displace 1–2 L of water with no pressure head in an inverted graduated cylinder, nested within a larger cylinder), and then placing the remaining MFMs and MFC in series with the calibrated MFC and recording their outputs at a series of controlled flow rates. We placed a Li-Cor quantum sensor (Li-190R) at various distances from each chamber’s light source to determine the leaf-to-light distance required to produce a saturating PPFD of 1700 μmol m^−2^ s^−1^ at the leaf surface.

We measured the leaf area enclosed in each chamber by marking the leaf at the external gasket margins, removing the leaf and taking a digital photograph of the enclosed leaf segment over a template representing the chamber and its gaskets, binarizing these images in ImageJ to produce an image with distinct white and black areas representing chamber areas with and without leaf, respectively, quantifying the % black pixels in the entire chamber area and multiplying the result by total chamber area (55 cm^2^).

To ensure that calculated *A*_max_ was not influenced by diffusion of CO_2_ across chamber gaskets, we recorded *A* with no leaf in the chamber and chamber CO_2_ concentrations in the typical range for this study (~ 4800–5000 ppm).

### System cost

The total cost of OCTOflux was approximately USD $31,000 (Table 1), 60% of which was the IRGA (USD $18,311), and another 30% of which was the eight sample MFMs (@ USD $647), two MFCs (@ USD $820), a laptop computer (USD $1725) and the DAQ board (USD $1493). The only significant running cost was compressed air (approximately 0.7–1 G-size cylinders/day or roughly USD $14–20/day or ~ $0.13–$0.18 per measurement; this cost may differ among countries or regions).

**Table 1:** OCTOflux components and approximate costs in USD in 2016–2017

Item	Count	Unit cost	Total cost
Gas analyzer	1	18,311	18,311
Mass flow meters	8	647	5176
Mass flow controllers	2	820	1640
Laptop computer	1	1725	1725
Data acquisition board	1	1493	1493
Gas regulators	2	250	500
Hardware, materials	1	500	500
Tubing, fittings, valves	1	500	500
LED lights and regulators	8	56	450
Relay control board	1	443	443
Solenoid valves	18	17	306
Garden cart	1	150	150
Total			$31,193

### Temperature correction

OCTOflux does not include leaf temperature (*T*) control. To minimize temperature fluctuations, we operated the system in an air-conditioned workshop; leaf *T* in the OCTOflux chamber averaged 26.0 ± 1.7 °C (mean ± SD), and 80% of measurements were between 24.1 and 28.2 °C. To correct *A*_max_ values to a common temperature of 25 °C, we determined the relationship between *A*_max_ and *T* as follows. We measured *A*_max_ at three temperatures (21.1 ± 0.1, 26.1 ± 0.3 and 31.1 ± 0.05 °C) in each of 10 leaves, using a calibrated infrared gas analyser (GFS-3000; Heinz Walz GmbH, Effeltrich, Germany). For each leaf, we fitted the function *A*_max_(*T*) = *a*·exp(*b*·*T*) to the data, computed *A*_max25_ for that leaf as *a*·exp(*b*·25), and expressed each *A*_max_ value for that leaf relative to its *A*_max25_, as *A*_rel_ = *A*_max_(*T*)/*A*_max25_. We then compiled *A*_rel_ values across leaves for each temperature, fitted the function *A*_rel_(*T*) = *a′*·exp(*b′*·*T*) to them and used this function to infer *A*_max25_ for each observed value of *A*_max_ in the study.

### Plant material

Leaves were chosen haphazardly from among flag (first-rank) and penultimate (second-rank) leaves of wheat (*Triticum aestivum* L.) as part of a broader study. Each leaf was from a different genotype; the complete genotype list is given in Additional file 3. All plants were grown in the field at the University of Sydney’s IA Watson Grains Research Centre, Narrabri, NSW Australia (30.2743°S, 149.8093°E). Plants were sown in 2 × 6 m plots with five planting rows, and lanes were later mowed between adjacent ranges of plots, making each plot 2 × 4 m at the time of measurement. Most plants were approximately at anthesis, and ranged in phenological stage from Zadok stage 57–71 (ear three quarters emerged to kernel water ripe, respectively).

Plants were haphazardly selected from the middle three planting rows at least 0.5 m from the end of each plot and cut at the base, immediately recut under distilled water and returned to the laboratory for measurement (approx. 2 km from the field), and then dark-acclimated for 0–60 min before measurement. Stomatal conductance typically exhibits a transient decline following plant or leaf excision in water, followed by a steady-state increase; analogous transients following excision in air averaged 6.7 min in duration across 20 species [14], and 2.0 min in the grass *Hordeum vulgare* (barley), which is closely related to wheat. These transients did not affect our estimates of *A*_max_ in the present study, because at least 20 min passed between excision and the final *A*_max_ measurement, but more importantly because leaves experienced saturating CO_2_, negating the impact of variations in stomatal conductance.

## Additional files

**Additional file 1.** Microsoft Excel file with VBA code that controls the OCTOflux system.

**Additional file 2.** VB.net code for DLL functions that handle communications between VBA and peripherals.

**Additional file 3.** List of genotypes used in trial application of the OCTOflux system.
