# Supplementary material for: A multiplexed gas exchange system for increased throughput of photosynthetic capacity measurements
Source: Plant Methods. 2018 Sep 11;14:80. doi: 10.1186/s13007-018-0347-y (PMC6131801; doi:10.1186/s13007-018-0347-y)
Supplement: Supplementary file 2 — Additional file 2. VB.net code for DLL functions that handle communications between VBA and peripherals. [file 13007_2018_347_MOESM2_ESM.pdf]

Supporting Information File S2

William T. Salter et al. ("A multiplexed gas exchange system for increased throughput of photosynthetic capacity")

Contents: VB.net code for functions in DLL that handles communications between the OCTOflux Excel/VBA program and a Measurement Computing Corporation (MCC) analog input/output board (model USB-2416), an MCC relay control board (USB-ERB24) and a Li-Cor Li-7000 infrared gas analyzer. Comments are shown in red text

```
Public Class octoIO

    Private licor As New IO.Ports.SerialPort 'create port object representing serial connection to Li-7000
    Private licortext As String

    ' function to read in analog outputs from the USB-2416 board
    Public Function from2416(Optional ByVal boardnum As Integer = 1) As Double()
        Dim ei As MccDaq.ErrorInfo
        Dim db2416 As MccDaq.MccBoard = New MccDaq.MccBoard(boardnum) 'create object representing the board
        Dim x32(28) As Int32
        Dim y(28) As Double
        Dim c As Integer
        Dim Options As Integer = 0

        'read thermocouple inputs (8 t/c, in channels 0,1,2,3,12,13,14,15)
        For c = 0 To 3
               ei = db2416.TIn(c, MccDaq.TempScale.Celsius, y(c), Options)
               If ei.Value <> MccDaq.ErrorInfo.ErrorCode.NoErrors Then x32(c) = -1000000000 'set return value to 1e-9 if error
        Next c
        For c = 12 To 15
               ei = db2416.TIn(c, MccDaq.TempScale.Celsius, y(c), Options)
               If ei.Value <> MccDaq.ErrorInfo.ErrorCode.NoErrors Then x32(c) = -1000000000
        Next c

        'read non-thermocouple analog inputs (channels 4-11, 16-27), then convert to engineering units (voltages)
        For c = 4 To 11
               ei = db2416.AIn32(c, MccDaq.Range.Bip5Volts, x32(c), Options)
               If ei.Value <> MccDaq.ErrorInfo.ErrorCode.NoErrors Then x32(c) = -1000000000

               ei = db2416.ToEngUnits32(MccDaq.Range.Bip5Volts, x32(c), y(c))
               If ei.Value <> MccDaq.ErrorInfo.ErrorCode.NoErrors Then y(c) = -20000000000.0
        Next c

        For c = 16 To 27
               ei = db2416.AIn32(c, MccDaq.Range.Bip5Volts, x32(c), Options)
               If ei.Value <> MccDaq.ErrorInfo.ErrorCode.NoErrors Then x32(c) = -1000000000

               ei = db2416.ToEngUnits32(MccDaq.Range.Bip5Volts, x32(c), y(c))
               If ei.Value <> MccDaq.ErrorInfo.ErrorCode.NoErrors Then y(c) = -20000000000.0
        Next c
```

```
    Return y
End Function

' send digital controls to relay control board to turn on/off relays that open or close solenoid valves
Public Function toERBdig(DIObit As Integer, OnOff As Integer, Optional ByVal boardnum As Integer = 0) As Integer
    Dim ei As MccDaq.ErrorInfo
    Dim dbERB As MccDaq.MccBoard = New MccDaq.MccBoard(boardnum)

    'configure this bit for output, then write 1 or 0 (OnOff) to the port
    ei = dbERB.DConfigBit(MccDaq.DigitalPortType.FirstPortA, DIObit, MccDaq.DigitalPortDirection.DigitalOut)
    ei = dbERB.DBitOut(MccDaq.DigitalPortType.FirstPortA, DIObit, OnOff)

    Return 1
End Function


' tell USB-2416 to send analog output voltage "AOvalue" to mass flow controller whose setpoint voltage is in channel "AOchannel"
Public Function to2416ana(AOchannel As Integer, AOvalue As Short, Optional ByVal boardnum As Integer = 1) As Integer
    Dim ei As MccDaq.ErrorInfo
    Dim db2416 As MccDaq.MccBoard = New MccDaq.MccBoard(boardnum)

    ei = db2416.AOut(AOchannel, MccDaq.Range.Uni10Volts, AOvalue)

    Return 1
End Function

' read in data string from Li-7000
Public Function fromLICOR(ComPort As String, delayms As Integer) As String
    Dim x As String
    With licor
        .PortName = ComPort
        .BaudRate = 115200
        .DataBits = 8
        .Parity = Parity.None
        .StopBits = StopBits.One
        .Handshake = Handshake.XOnXOff
        .ReadTimeout = 500
    End With

    Try
        licor.Open()
    Catch ex As Exception
    End Try

    licor.WriteLine("(RS232(Poll Now))")
    licor.DiscardInBuffer()
    licor.WriteLine("(RS232(Poll Now))")
    Thread.Sleep(delayms)
```

```
        x = licor.ReadExisting
        licor.Close()
        Return x
    End Function

    ' send text string to Li-7000
    Public Sub toLICOR(ComPort As String, message As String)
        With licor
            .PortName = ComPort
            .BaudRate = 115200
            .DataBits = 8
            .Parity = Parity.None
            .StopBits = StopBits.One
            .Handshake = Handshake.XOnXOff
            .ReadTimeout = 500
        End With

        Try
            licor.Open()
        Catch ex As Exception
        End Try

        licor.WriteLine(message)
        licor.DiscardInBuffer()
        licor.Close()
    End Sub

End Class
```
