# Supplementary material for: A multiplexed gas exchange system for increased throughput of photosynthetic capacity measurements
Source: Plant Methods. 2018 Sep 11;14:80. doi: 10.1186/s13007-018-0347-y (PMC6131801; doi:10.1186/s13007-018-0347-y)
Supplement: Supplementary file 3 — Additional file 3. List of genotypes used in trial application of the OCTOflux system. [file 13007_2018_347_MOESM3_ESM.pdf]

Supporting Information File S3

William T. Salter et al. ("A multiplexed gas exchange system for increased throughput of photosynthetic capacity")

Contents: list of genotypes used in test of OCTOflux system described in main manuscript.

genotype #	name/identifier	pedigree
2	DBW16	DBW16
4	PBW502	PBW502
6	SOKOLL	SOKOLL
7	MACE	MACE
9	WAXWING*2/KIRITATI	WAXWING*2/KIRITATI
10	2.49/CUNNINGHAM/KENNEDY	2.49/CUNNINGHAM/KENNEDY
11	PBI09C016-BC-DH4	2.49/CUNNINGHAM/KENNEDY/3/2.49/CUNNINGHAM/KENNEDY /2/ 35880 M C18644
12	PBI09C018-BC-DH2	2.49/CUNNINGHAM/KENNEDY/3/2.49/CUNNINGHAM/KENNEDY /2/ 35883 M500110
14	PBI09C004-BC-DH23	BERKUT/2/BERKUT / 35883 M500110
15	PBI09C004-BC-DH24	BERKUT/2/BERKUT / 35883 M500110
16	PBI09C004-BC-DH74	BERKUT/2/BERKUT / 35883 M500110
18	PBI09C004-BC-DH78	BERKUT/2/BERKUT / 35883 M500110
19	PBI09C004-BC-DH106	BERKUT/2/BERKUT / 35883 M500110
20	PBI09C004-BC-DH117	BERKUT/2/BERKUT / 35883 M500110
21	PBI09C004-BC-DH118	BERKUT/2/BERKUT / 35883 M500110
22	PBI09C004-BC-DH1	BERKUT/2/BERKUT / 35883 M500110
23	PBI09C004-BC-DH51	BERKUT/2/BERKUT / 35883 M500110
25	PBI09C001-BC-DH9	BERKUT/2/BERKUT / 35880 M C18644
26	PBI09C001-BC-DH58	BERKUT/2/BERKUT / 35880 M C18644
27	PBI09C001-BC-DH64	BERKUT/2/BERKUT / 35880 M C18644
29	PBI09C001-BC-DH46	BERKUT/2/BERKUT / 35880 M C18644
30	PBI09C045-BC-DH7	DBW16 /2/DBW16 / 18341 KC75
31	PBI09C045-BC-DH13	DBW16 /2/DBW16 / 18341 KC75

32	PBI09C045-BC-DH15	DBW16 /2/DBW16 / 18341 KC75
33	PBI09C045-BC-DH28	DBW16 /2/DBW16 / 18341 KC75
34	PBI09C045-BC-DH30	DBW16 /2/DBW16 / 18341 KC75
35	PBI09C045-BC-DH31	DBW16 /2/DBW16 / 18341 KC75
36	PBI09C043-BC-DH21	DBW16 /2/DBW16 / 21758 KC75
39	PBI09C043-BC-DH28	DBW16 /2/DBW16 / 21758 KC75
43	PBI09C049-BC-DH5	DBW17 /2/DBW17 / 19385 KC79
44	PBI09C049-BC-DH6	DBW17 /2/DBW17 / 19385 KC80
45	PBI09C048-BC-DH11	DBW17 /2/DBW17 / 21758 KC75
49	PBI09C048-BC-DH35	DBW17 /2/DBW17 / 21758 KC75
50	PBI09C048-BC-DH36	DBW17 /2/DBW17 / 21758 KC75
51	PBI09C047-BC-0C-1N-99N	DBW17//DBW17 / 18293 KC75
52	PBI09C035-BC-DH6	PBW502 /2/PBW502 / 19385 KC75
53	PBI09C035-BC-DH31	PBW502 /2/PBW502 / 19385 KC75
55	PBI09C035-BC-DH7	PBW502 /2/PBW502 / 19385 KC75
56	PBI09C035-BC-DH11	PBW502 /2/PBW502 / 19385 KC75
58	PBI09C035-BC-DH21	PBW502 /2/PBW502 / 19385 KC75
59	PBI09C035-BC-DH25	PBW502 /2/PBW502 / 19385 KC75
60	PBI09C035-BC-DH26	PBW502 /2/PBW502 / 19385 KC75
61	PBI09C035-BC-DH28	PBW502 /2/PBW502 / 19385 KC75
62	PBI09C035-BC-DH41	PBW502 /2/PBW502 / 19385 KC75
64	PBI09C035-BC-DH22	PBW502 /2/PBW502 / 19385 KC75
65	PBI09C034-BC-DH23	PBW502 /2/PBW502 / 21758 KC75
66	PBI09C034-BC-DH29	PBW502 /2/PBW502 / 21758 KC75
67	PBI09C034-BC-DH30	PBW502 /2/PBW502 / 21758 KC75
68	PBI09C034-BC-DH33	PBW502 /2/PBW502 / 21758 KC75
69	PBI09C034-BC-DH34	PBW502 /2/PBW502 / 21758 KC75
71	PBI09C034-BC-DH21	PBW502 /2/PBW502 / 21758 KC75
73	PBI09C034-BC-DH17	PBW502 /2/PBW502 / 21758 KC75

75	PBI09C038-BC-DH4	PBW550 /2/PBW550 / 18293 KC75
78	PBI09C038-BC-DH9	PBW550 /2/PBW550 / 18293 KC75
79	PBI09C038-BC-DH17	PBW550 /2/PBW550 / 18293 KC75
80	PBI09C038-BC-DH1	PBW550 /2/PBW550 / 18293 KC75
81	PBI09C038-BC-DH10	PBW550 /2/PBW550 / 18293 KC75
82	PBI09C038-BC-DH22	PBW550 /2/PBW550 / 18293 KC75
83	PBI09C038-BC-DH21	PBW550 /2/PBW550 / 18293 KC75
84	PBI09C039-BC-DH9	PBW550 /2/PBW550 / 18343 KC75
86	PBI09C039-BC-DH47	PBW550 /2/PBW550 / 18343 KC75
88	PBI09C039-BC-DH63	PBW550 /2/PBW550 / 18343 KC75
89	PBI09C039-BC-DH81	PBW550 /2/PBW550 / 18343 KC75
90	PBI09C010-BC-DH1	SOKOLL/2/SOKOLL / 35879 M C18643
93	PBI09C010-BC-DH9	SOKOLL/2/SOKOLL / 35879 M C18643
95	PBI09C010-BC-DH15	SOKOLL/2/SOKOLL / 35879 M C18643
96	PBI09C010-BC-DH18	SOKOLL/2/SOKOLL / 35879 M C18643
97	PBI09C010-BC-DH19	SOKOLL/2/SOKOLL / 35879 M C18643
98	PBI09C008-BC-DH1	SOKOLL/2/SOKOLL / 35883 M500110
99	PBI09C008-BC-DH8	SOKOLL/2/SOKOLL / 35883 M500110
102	PBI09C008-BC-DH31	SOKOLL/2/SOKOLL / 35883 M500110
104	PBI09C008-BC-DH35	SOKOLL/2/SOKOLL / 35883 M500110
105	PBI09C008-BC-DH7	SOKOLL/2/SOKOLL / 35883 M500110
106	PBI09C008-BC-DH17	SOKOLL/2/SOKOLL / 35883 M500110
112	PBI09C009-BC-DH84	SOKOLL/2/SOKOLL / 35888 M 500132
113	PBI09C009-BC-DH86	SOKOLL/2/SOKOLL / 35888 M 500132
115	PBI09C009-BC-DH30	SOKOLL/2/SOKOLL / 35888 M 500132
116	PBI09C009-BC-DH52	SOKOLL/2/SOKOLL / 35888 M 500132
118	PBI09C009-BC-DH16	SOKOLL/2/SOKOLL / 35888 M 500132
119	PBI09C028-BC-DH33	WAXWING*2/KIRITATI /3/WAXWING*2/KIRITATI /2/ 35880 M C18644
121	PBI09C028-BC-DH18	WAXWING*2/KIRITATI /3/WAXWING*2/KIRITATI /2/ 35880 M C18644

122	PBI09C028-BC-DH2	WAXWING*2/KIRITATI /3/WAXWING*2/KIRITATI /2/ 35880 M C18644
123	PBI09C028-BC-DH3	WAXWING*2/KIRITATI /3/WAXWING*2/KIRITATI /2/ 35880 M C18644
124	PBI09C028-BC-DH5	WAXWING*2/KIRITATI /3/WAXWING*2/KIRITATI /2/ 35880 M C18644
125	PBI09C028-BC-DH7	WAXWING*2/KIRITATI /3/WAXWING*2/KIRITATI /2/ 35880 M C18644
128	PBI09C028-BC-DH37	WAXWING*2/KIRITATI /3/WAXWING*2/KIRITATI /2/ 35880 M C18644
130	PBI09C028-BC-DH54	WAXWING*2/KIRITATI /3/WAXWING*2/KIRITATI /2/ 35880 M C18644
131	PBI09C026-BC-DH4	WAXWING*2/KIRITATI /3/WAXWING*2/KIRITATI /2/ 35888 M 500132
133	PBI09C026-BC-DH121	WAXWING*2/KIRITATI /3/WAXWING*2/KIRITATI /2/ 35888 M 500132
134	PBI09C026-BC-DH31	WAXWING*2/KIRITATI /3/WAXWING*2/KIRITATI /2/ 35888 M 500132
135	PBI09C026-BC-DH65	WAXWING*2/KIRITATI /3/WAXWING*2/KIRITATI /2/ 35888 M 500132
137	PBI09C026-BC-DH71	WAXWING*2/KIRITATI /3/WAXWING*2/KIRITATI /2/ 35888 M 500132
138	PBI09C026-BC-DH73	WAXWING*2/KIRITATI /3/WAXWING*2/KIRITATI /2/ 35888 M 500132
139	PBI09C026-BC-DH88	WAXWING*2/KIRITATI /3/WAXWING*2/KIRITATI /2/ 35888 M 500132
142	PBI09C026-BC-DH114	WAXWING*2/KIRITATI /3/WAXWING*2/KIRITATI /2/ 35888 M 500132
143	31 : ZWW10	BABAX/LR42//BABAX/3/BABAX/LR42//BABAX/4/T.DICOCCON PI94625/AE.SQUARROSA (372)//3*PASTOR/5/T.DICOCCON PI94625/AE.SQUARROSA (372)//3*PASTOR
145	19 : ZIZ10	CBRD-3/STORK X DICOCCOIDES
146	7 : ZWW11	D67.2/P66.270//AE.SQUARROSA (320)/3/CUNNINGHAM/4/VORB
147	238 : ZWB13	FALCIN/AE.SQUARROSA (312)/3/THB/CEP7780//SHA4/LIRA/4/FRET2/5/MUU/6/MILAN/KAUZ//DHARWAR DRY/3/BAV92
148	FILIN/IRENA/5/…./6/BERKUT	FILIN/IRENA/5/CNDO/R143//ENTE/MEXI_2/3/AEGILOPS SQUARROSA (TAUS)/4/WEAVER/6/BERKUT
149	135 : ZWW10	GOUBARA-1/2*SOKOLL
150	27 : ZIZ13	KAUZ/PASTOR/3/ALTAR 84/AEGILOPS SQUARROSA (TAUS)//OPATA
151	28 : ZIZ13	KAUZ/PASTOR/3/ALTAR 84/AEGILOPS SQUARROSA (TAUS)//OPATA
153	334 : ZWB13	SOKOLL/3/PASTOR//HXL7573/2*BAU/4/PARUS/PASTOR
154	315 : ZWB13	SOKOLL/WBLL1/4/ATTILA/3/WEAVER*2/TSC//WEAVER
155	306 : ZWB13	VEE/MJI//2*TUI/3/PASTOR/4/BERKUT/11/CROC_1/AE.SQUARROSA (213)//PGO/10/ATTILA*2/9/KT/BAGE//FN/U/3/BZA/4/TRM/5/ALDAN/6/SERI/7/VEE#10/8/OPATA
158	ACIAR09PBI C27-0C-0N-13N	DBW16/ANNUELLO
160	ACIAR09PBI C04-16C-DH17	PBW550//C80.1/*2Batavia

161	ACIAR09PBI C04-23C-DH7	PBW550//C80.1/*2Batavia
163	ACIAR09PBI C08-0C-0N-11N	PBW550/LANG
164	ACIAR09PBI C06-0C-0N-2N	PBW550/PAVON
167	89 : ZIZ13	ATTILA-7/KATILA-12
169	92 : ZWB13	MUU/FRNCLN//FRANCOLIN #1
171	171 : ZWB13	QUAIU/BECARD//BECARD
176	35 : ZWB14	ROLF07*2/PAURAQ
177	43 : ZWB14	SUP152/2*DANPHE #1
179	103 : ZWB14	PAURAQ/3/KIRITATI//PRL/2*PASTOR/4/PAURAQUE #1
180	142 : ZWB14	PRL/2*PASTOR/4/CHOIX/STAR/3/HE1/3*CNO79//2*SERI/5/2*PAURAQUE #1
183	SPITFIRE	SPITFIRE
185	39: ZIG14	02W50807/4/PFAU/SERI.1B//AMAD/3/WAXWING
187	18: CWA15	HUBARA-7/4/CHEN/AEGILOPS SQUARROSA (TAUS)//BCN/3/KAUZ
188	71: CWA15	KAUZ/SAMAR-15//RDWG/MILAN
189	91: CWA15	JAWAHIR-1/GIRWILL-5
190	SUN691I	SUN691I
192	F946	
193	F589	
194	F561	
195	F1036	
196	F550	
199	PASCAL	
202	F1572	
205	BOLAC	
206	BEAUFORT	
207	GREGORY	
208	F1222	
210	F808	
212	F883	

213	F656	
214	F1181	
216	F1431	
218	F406	
220	F233	
221	F1178	
224	F1536	
226	F1303	
227	F1501	
229	F658	
230	F764	
233	F235	
234	F455	
237	F979	
238	F788	
239	F907	
241	F1265	
242	F1579	
243	F1587	
244	F836	
246	F1584	
247	C1061+	
248	C1061-	
250	C10710-	
